# Expression of Concern: Long non-coding RNA linc-ITGB1 promotes cell proliferation, migration and invasion in human hepatoma carcinoma by upregulating ROCK1

**DOI:** 10.1042/BSR-20181289_EOC

**Published:** 2021-04-19

**Keywords:** hepatoma carcinoma, invasion, Linc-ITGB1, migration, proliferation, ROCK1

The Editorial Office has been made aware of potential issues surrounding the scientific validity of this paper, hence has issued an expression of concern to notify readers whilst the Editorial Office investigates.

